# Selective cytotoxicity of indirect nonequilibrium atmospheric pressure plasma against ovarian clear-cell carcinoma

**DOI:** 10.1186/2193-1801-3-398

**Published:** 2014-07-31

**Authors:** Fumi Utsumi, Hiroaki Kajiyama, Kae Nakamura, Hiromasa Tanaka, Masaru Hori, Fumitaka Kikkawa

**Affiliations:** Department of Obstetrics and Gynecology, Nagoya University Graduate School of Medicine, Tsuruma-cho 65, Showa-ku, Nagoya, 466-8550 Japan; Department of Electrical Engineering and Computer Science, Graduate School of Engineering, Nagoya University, Furo-cho, Chikusa-ku, Nagoya, 464-8603 Japan

**Keywords:** Nonequilibrium atmospheric pressure plasma, Epithelial ovarian cancer (EOC), Clear-cell carcinoma, Chemoresistance, Apoptosis, Selective cytotoxicity

## Abstract

Ovarian clear cell carcinoma (CCC) is a histological type of epithelial ovarian cancer that is less responsive to chemotherapy and associated with a poorer prognosis than serous and endometrioid carcinoma. Non-thermal atmospheric pressure plasma which produces reactive species has recently led to an explosion of research in plasma medicine. Plasma treatment can be applied to cancer treatment to induce apoptosis and tumor growth arrest. Furthermore, recent studies have shown that a medium exposed to plasma also has an anti-proliferative effect against cancer in the absence of direct exposure to plasma. In this study, we confirmed whether this indirect plasma has an anti-tumor effect against CCC, and investigated whether this efficacy is selective for cancer cells. Non-thermal atmospheric pressure plasma induced apoptosis in CCC cells, while human peritoneal mesothelial cells remained viable. Non-thermal atmospheric pressure plasma exhibits selective cytotoxicity against CCC cells which are resistant to chemotherapy.

## Background

Epithelial ovarian carcinoma (EOC) is the most frequent cause of gynecological cancer-related death in women in Western countries. Ovarian clear-cell carcinoma (CCC), a subtype of EOC, is relatively less sensitive to chemotherapy, and is therefore recognized as refractory ovarian cancer (Kajiyama et al. 2012). Even though a combination of carboplatin and paclitaxel have been established as standard therapy for ovarian cancer (McGuire et al. 1996), CCC shows lower response rates (Itamochi et al. 2008; Anglesio et al. 2011), while serous adenocarcinoma and endometrioid adenocarcinoma respond well to this regimen. Moreover, the incidence of CCC has been increasing, and it is now estimated to be more than 20% in Japan, whereas that in Europe is reportedly 5-6% (Anglesio et al. 2011). Although various additional molecular-targeting therapies, including anti-angiogenic agents and cancer vaccine treatment, have been investigated in order to overcome such chemoresistance, the effect of such treatment is not satisfactory, and no treatment has been established for this histological subtype of EOC.

Plasma is defined as ionized gas containing energy particles including electrons, ions, neutral atoms, and molecules such as free radicals and electronically excited atoms. Since it has become possible to generate plasma at room temperature and atmospheric pressure due to the technical developments, plasma has been gaining interest in medical field as well as industrial fields. In medical field plasma has been studied and applied for sterilization (Klampfl et al. 2012), implants (Coelho et al. 2012), blood coagulation (Bergler et al. 2001), and wound healing (Isbary et al. 2012). Additionally, it had been demonstrated that plasma can induce apoptosis in cancerous cells (Fridman et al. 2007; Keidar et al. 2011; Kim et al. 2010a; Kalghatgi et al. 2011), and it is expected to provide an alternative for cancer treatment. What is required for anti-neoplastic agents is to have selective cytotoxicity against cancer cells with minimized side effects on normal cells and have appropriate drug delivery system that allows the agents to reach the targeted tumor. Some researchers have reported the therapeutic potential of the selective cytotoxicity of plasma (Keidar et al. 2011; Kim et al. 2009; Tanaka et al. 2011). In our previous study, we also confirmed the selectivity of plasma against EOC (Iseki et al. 2012; Utsumi et al. 2013).

Although the mechanism behind this selective cytotoxicity of plasma is not fully understood, plasma may generate various reactive oxygen species (ROS), such as superoxide (O_2_^▪-^) hydroxyl (OH^▪^), hydrogen peroxide (H_2_O_2_), singlet oxygen (^1^O_2-_), ozone (O_3_), leading to peroxidation of the lipid double membrane and resulting in apoptosis (Kalghatgi et al. 2011; Vandamme et al. 2012; Ishaq et al. 2014; Yan et al. 2012a). Cancer cells generate more intrinsic ROS than normal cells due to their high metabolic activity. Therefore, cancer cells may be more vulnerable to ROS-inducing reagents, like some chemotherapeutic drugs. Furthermore, several ROS generated by plasma have also been detected in the culture medium during exposure to plasma, called indirect plasma or plasma-activated medium, and this has been applied to cancer treatment. This method, in which cells are exposed to some liquid exposed to plasma beforehand, has been demonstrated to exhibit an anti-tumor effect comparable to direct plasma (Kalghatgi et al. 2011; Tanaka et al. 2011; Ryu et al. 2013). Considering the well-known characteristic of EOC disseminating throughout the peritoneal cavity, indirect plasma is suitable for intraperitoneal administration.

In this study, using indirect plasma in the form of nonequilibrium atmospheric pressure plasma (NEAPP), which is a type of non-thermal atmospheric pressure plasma, we demonstrated selective cytotoxicity against CCC cells, which are less sensitive to chemotherapy compared to normal cells. Taking intraperitoneal administration into account, we used human omental peritoneal mesothelial cells as normal cells.

## Results

### Effect of NEAPP-AM on clear cell carcinoma cell line

**Figure 1:**
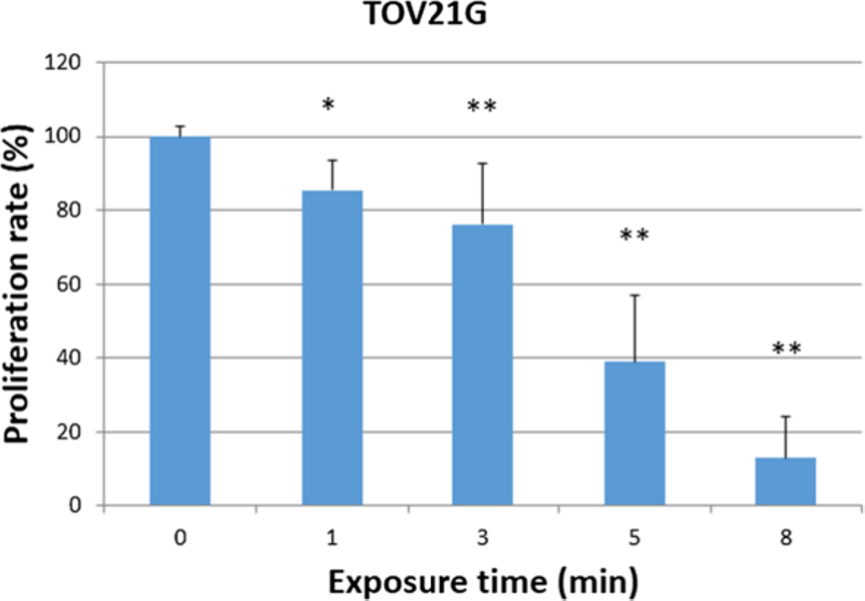
**The effect of NEAPP-AM on cell viability.** Viability of TOV21G ovarian clear cell carcinoma cells treated with NEAPP-activated medium (NEAPP-AM), as measured by the cell viability assay. TOV21G cells were plated in 96-well plates and incubated at 37°C with 5% CO2. After 24 hrs, the culture medium was replaced with NEAPP-AM and the cells were incubated for another 24 hrs. The cell proliferation rate was evaluated relative to the control with the cell viability assay. Each column represents the mean, and the bars show SD. Assays were performed in triplicate. *P < 0.05, **P < 0.01 versus control.

**Figure 2:**
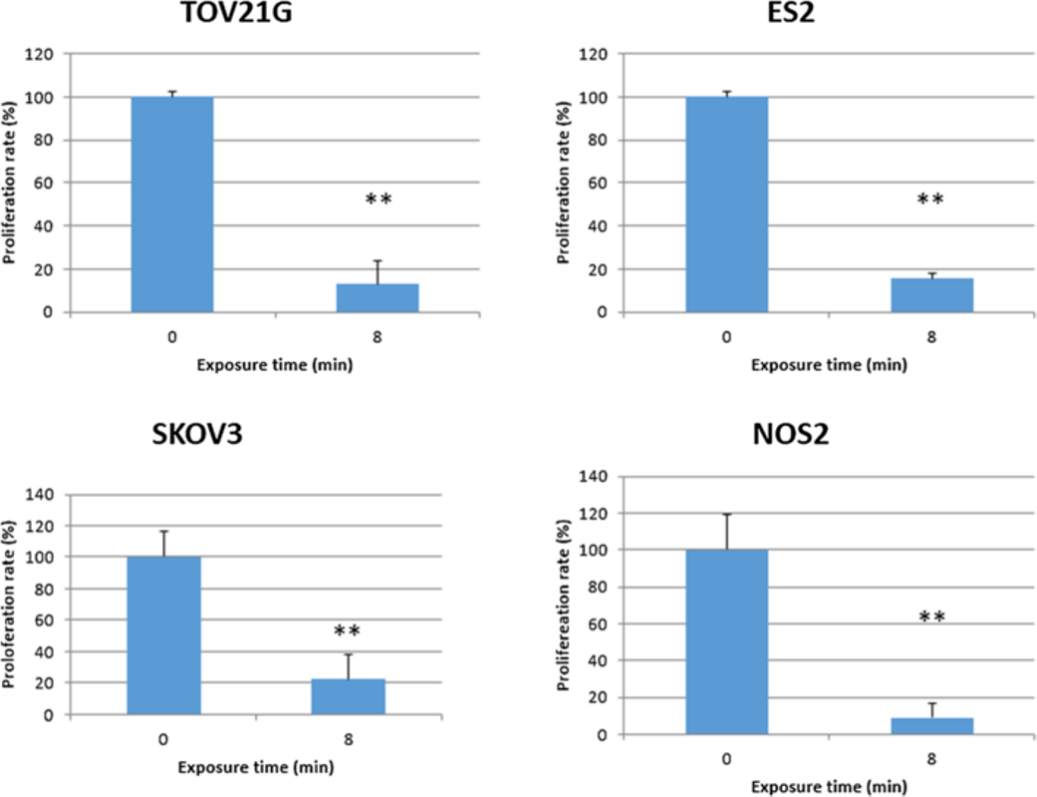
**Systematic analysis of the effect of NEAPP-AM on some EOC cell lines.** ES-2, SKOV3 and NOS2 cells were treated with NEAPP-AM-8. Cell viability was determined 24 hrs after treatment with the cell viability assay. Cell viability of treated cells was normalized to the control. *P < 0.05, **P < 0.01 versus control.

We first evaluated the anti-tumor effect of NEAPP-AM on the growth of the CCC cell line using the cell viability assay. Figure 1 shows the cell viability of TOV21G cells exposed to NEAPP-AM for 24 hrs, which was prepared beforehand by exposure to NEAPP for the indicated time. The viability decreased by approximately 61% when cells were treated with NEAPP-AM-5, and 87% after being treated with NEAPP-AM-8 compared to non-NEAPP-exposed medium treatment (*P* <0.01). These findings suggest that the NEAPP-AM treatment also has an anti-tumor effect on CCC cells. Furthermore, we confirmed the anti-tumor effect of NEAPP-AM on other EOC cell lines (ES-2, SKOV3, and NOS2). Figure 2 shows the cell viabilities of these cells exposed to NEAPP-AM-8 for 24 hrs. Significant cytotoxicity was demonstrated in every EOC cell line.

### Morphological changes observed in EOC cell lines induced by NEAPP-AM

**Figure 3:**
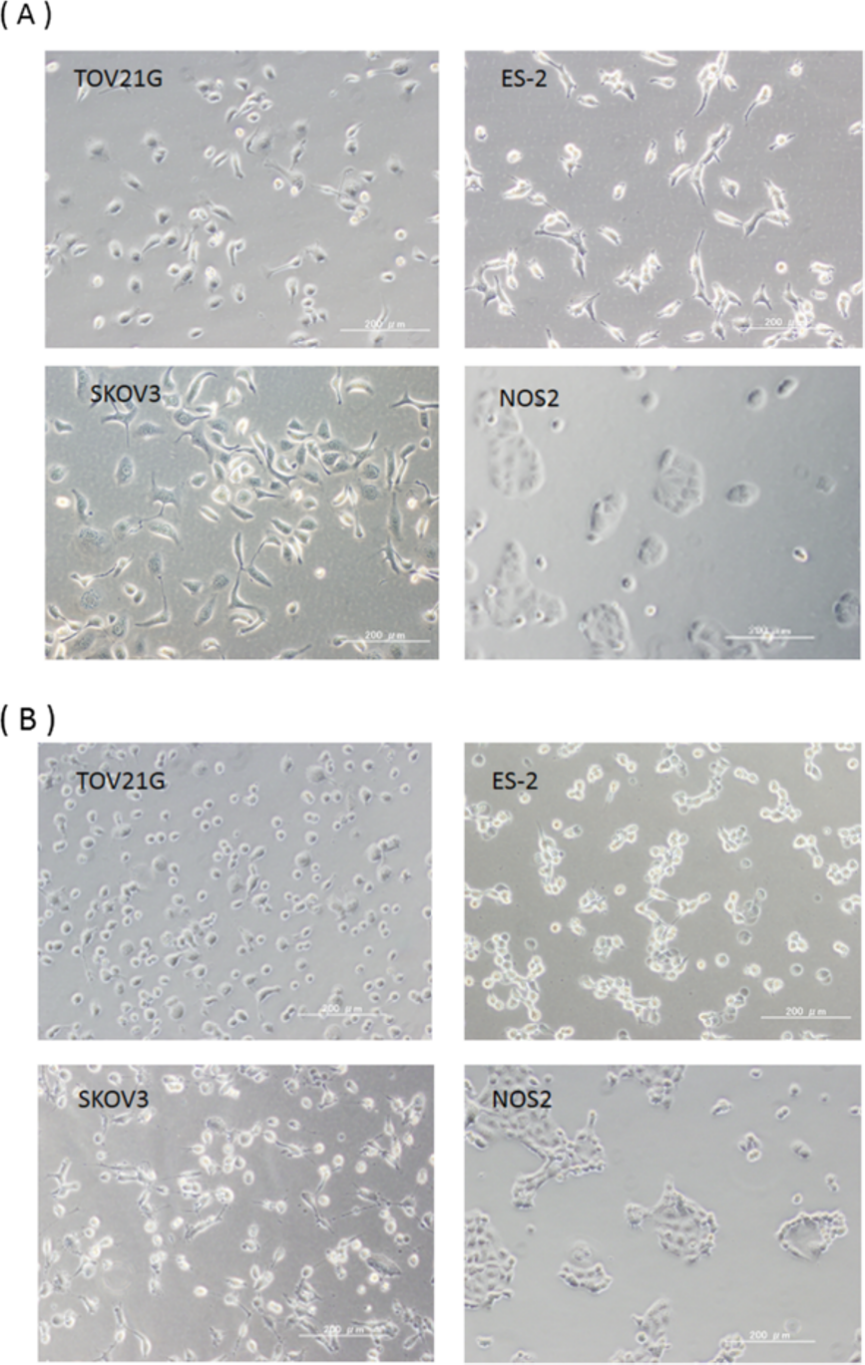
**The morphological changes of EOC cells after NEAPP-AM treatment.** ***A***: Representative images of untreated EOC cells (TOV21G, ES-2, SKOV3, and NOS2). ***B***: EOC cells (TOV21G, ES-2, SKOV3, and NOS2) treated with NEAPP-AM for 6 hrs.

Morphological investigations of EOC cells exposed to NEAPP-AM were performed. Images of untreated EOC cells are shown in (Figure 3A). The cells treated with NEAPP-AM (15) -5 were observed 6 hrs after treatment and displayed changes, such as shrinking, rounding up, and detachment from dishes which were typical of apoptosis (Figure 3B).

### Role of ROS and attenuated and enhanced NEAPP-AM effect with NAC and BSO

**Figure 4:**
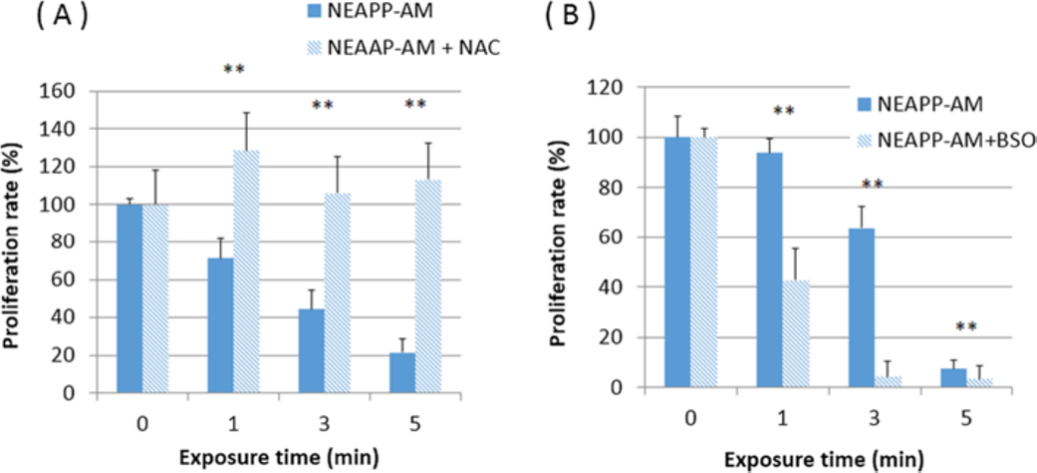
**Role of ROS and attenuated and enhanced NEAPP-AM sensitivity with NAC and BSO.** ***A, B***: Influence of intracellular ROS modulation by NAC and BSO on NEAPP-AM-induced cell death. **A**: TOV21G cells were pretreated with NAC (4 mM) **(A)** or BSO (2 mM) **(B)** for 1 hr and then exposed to NEAPP-AM with NAC or BSO for an additional 24 hrs. The cell viability assay was used for evaluation. Each column represents the mean and the bars are SD. Data are representative of at least three independent experiments. *P < 0.05, **P < 0.01 versus control without NAC treatment.

Subsequently, we examined whether ROS produced by NEAPP are responsible for the anti-tumor effects on CCC cells as well as serous EOC cells in tests we previously performed. To investigate the role of ROS in NEAPP-AM treatment, TOV21G cells were pre-incubated with NAC or BSO, followed by the addition of NEAPP-AM for the indicated exposure times. The viability of TOV21G cells was assayed 24 hrs after NEAPP-AM treatment using the cell viability assay. As shown in Figure 4, the growth-inhibitory effects of NEAPP-AM were completely blocked on being pretreatment and co-incubation with NAC (Figure 4A). In contrast, BSO (Figure 4B) with NEAPP-AM decreased cell viability; especially, BSO with NEAPP-AM-1, -3 led to a significant decrease, compared to the control (NEAPP-AM treatment alone) (*P* <0.01). These results suggest that ROS in cells produced by NEAPP-AM play a critical role in anti-tumor effects against EOC cells.

### NEAPP-AM-induced apoptosis in TOV21G and SKOV3

**Figure 5:**
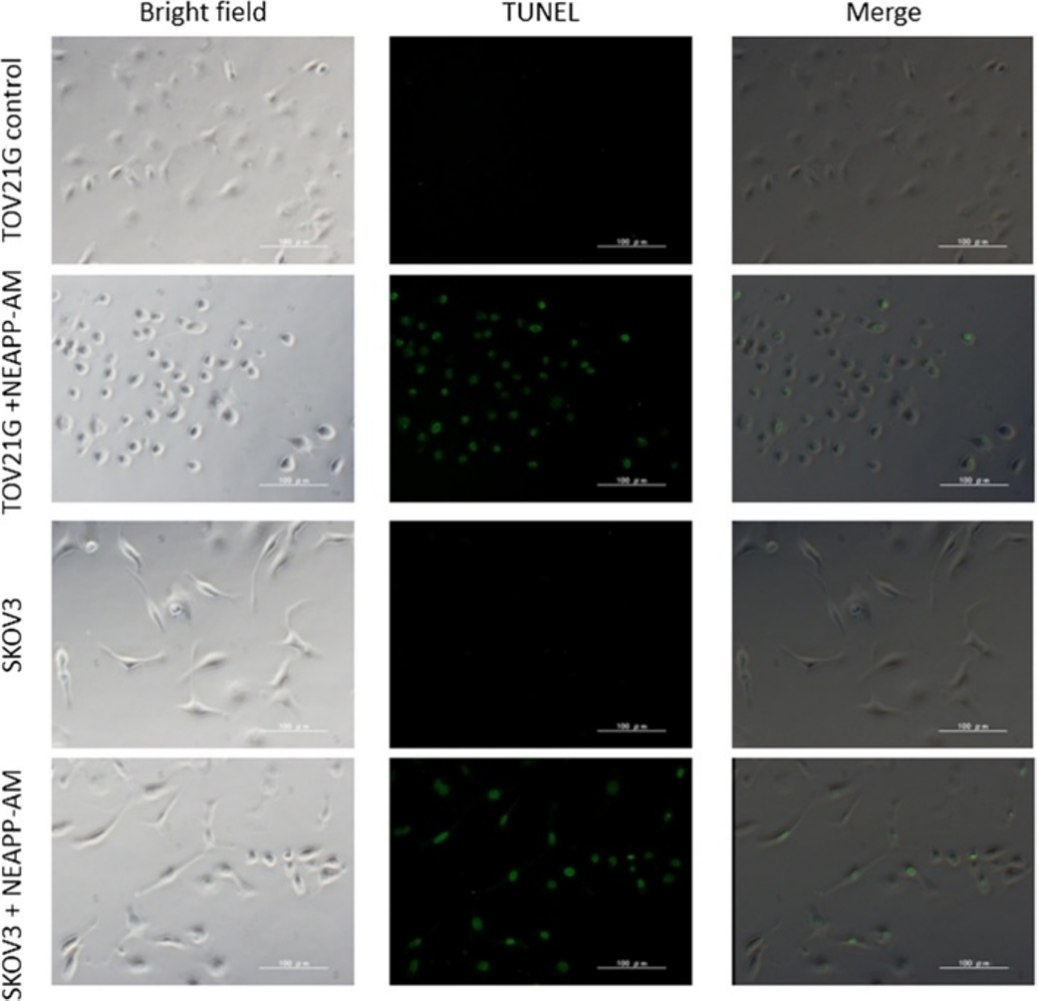
**TUNEL assay.** The TUNEL assay was performed to detect apoptosis in TOV21G and SKOV3 cells induced by NEAPP-AM. TOV21G and SKOV3 cells were treated with NEAPP-AM or control medium. Cells were incubated for 4 hrs, then fixed, and the TUNEL assay was performed.

Subsequently, we assessed whether the cytotoxic effect of NEAPP-AM against TOV21G and SKOV3 was associated with the induction of apoptosis by TUNEL assay. To identify inner nucleosomal DNA strand breaks, a characteristic feature of apoptosis, TUNEL staining was performed at 4 hrs after NEAPP-AM, treatment, as described in Materials and Methods. Compared with control cells, both TOV21G and SKOV3 cells treated with NEAPP-AM showed more TUNEL-positive staining cells, indicating that NEAPP-AM induced apoptosis in both cell lines (Figure 5).

### Selective cytotoxic effect of NEAPP-AM on TOV21G cells compared with OHFC and HPMC

**Figure 6:**
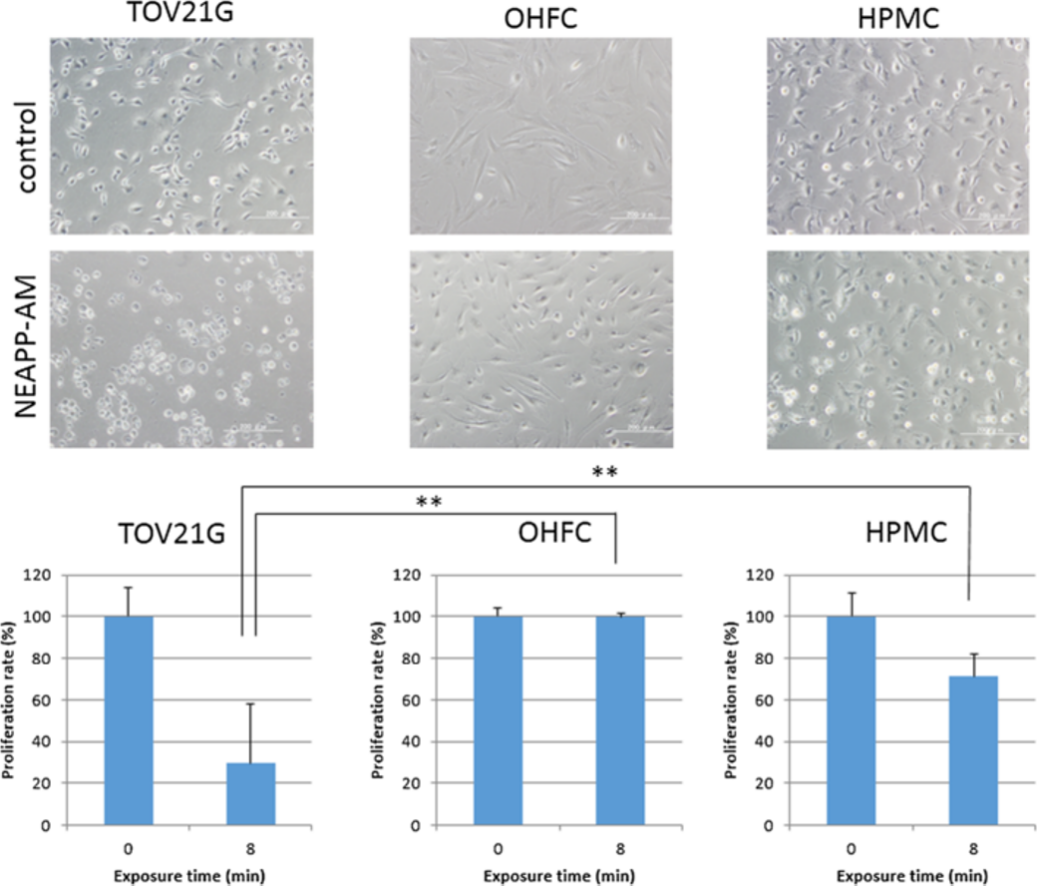
**Selective cytotoxic effect of NEAPP-AM on TOV21G cells compared with OHFC and HPMC.** Cell viability of TOV21G, OHFC, and HPMC after being treated with NEAPP-AM-8 was measured by the cell viability assay. Cells were seeded in collagen-coated 96-well plates and incubated for 24 hrs, the culture medium was replaced with NEAPP-AM, and the cells were incubated for another 24 hrs. The cell proliferation rate was evaluated relative to the control with the cell viability assay. *P < 0.05, **P < 0.01 versus TOV21G treated with NEAPP-AM-8.

We finally examined whether NEAPP-AM has a selective anti-proliferative effect on cancer cells without causing adverse reactions in normal cells. TOV21G, OHFC, and HPMC cells were exposed to NEAPP-AM (25)-8 for 24 hrs and investigated with cell viability assay. As shown in Figure 6, the viabilities of TOV21G, OHFC, and HPMC after being treated with NEAPP-AM were 29, 100, and 71%, respectively [OHFC vs. TOV21G (*P* <0.01): HPMC vs. TOV21G (*P* < 0.01). Remarkably, the cytotoxic effects of NEAPP-AM on normal human peritoneal cells are less sensitive than on cancer cells.

## Discussion

Plasma has been used for a long time for industrial purposes. Recently, with the rapid advancement of devices that can generate plasma at atmospheric pressure, plasma has received much attention in medical fields. Many researchers have demonstrated plasma-induced apoptosis in cancer cells (Fridman et al. 2007; Yan et al. 2012b; Kim et al. 2010b), and some of them have applied this treatment to *in vivo* studies (Keidar et al. 2011; Vandamme et al. 2012; Vandamme et al. 2010). Although these studies have indicated plasma treatment also has anti-tumor effects *in vivo*, the application is often limited to skin cancer or a xenografted model because of the characteristics of the device. When direct plasma was applied to an intraperitoneal tumor, the treatment became very invasive, involving an abdominal operation (Brulle et al. 2012). So, we have been focusing on the indirect effect of plasma using NEAPP-AM in order to administer plasma for ovarian cancer as safely as possible. ROS or reactive nitrogen species generated by plasma have been shown to be transferred to the medium and exert anti-tumor effect comparable to direct plasma (Kalghatgi et al. 2011; Yan et al. 2012a; Ryu et al. 2013). We recently reported the anti-proliferative effects of NEAPP-AM against chemoresistant EOC cell lines derived from serous EOC cells *in vitro* and *in vivo* in a xenografted model (Utsumi et al. 2013). In this study, we used a CCC cell line which is naturally chemoresistant and often associated with a poor prognosis. Our experiments demonstrated a selective cytotoxic effect of NEAPP-AM on CCC, compared with normal human cells. Furthermore, the majority of patients with EOC have advanced intraperitoneal metastasis and dissemination at the time of diagnosis. Since it is important to target these disseminations without damaging surrounding normal cells, we selected peritoneal mesothelial and fibroblastic cells as normal cells.

Several mechanisms involved in drug resistance of CCC have been proposed, including decreased drug accumulation, increased drug detoxification, and increased DNA repair activity. However, the details underlying CCC’s resistance to chemotherapy have not been clarified. Itamochi et al. suggested that the lower tumor proliferation may contribute to its resistance to chemotherapy (Itamochi et al. 2008). Genetically, glutathione peroxidase (*GPx3*), glutaredoxin (*GLRX*), and superoxide dismutase (*SOD2*) were highly expressed in CCC tumors, and high levels of these antioxidant proteins may be associated, in part, with the chemoresistant phenotype of CCC (Schwartz et al. 2002). On the other hand, the anti-tumor effect of plasma has been suggested to be triggered through lipid peroxidation with an excessive amount of ROS generated by plasma. Apoptosis caused by lipid peroxidation has been related to the activity of p53, which is known as a tumor suppressor triggering cell-cycle arrest and apoptosis (Yan et al. 2012a; Tsuzuki et al. 2007). Xu Yan et al. examined mRNA expression, and revealed that cyclin B1 and Cdc2 are decreased at the transcriptional level after plasma treatment, while the expression of p21 Cdk inhibitor, as well as that of tumor suppressor p53, is enhanced (Yan et al. 2012b). This mechanical difference between chemotherapy and plasma treatment may contribute to the anti-proliferative effect of plasma against CCC.

Although, plasma may be successfully used for superficial lesions, including the stimulation of wound healing, sterilization of medical instruments, and removal of microscopic residual cancer, regarding the use of atmospheric pressure plasma as a treatment for cancer patients, there may be some limitations such as a lack of the infiltrating capability of plasma. The depth that can be penetrated by plasma is thought to be limited. We are trying to apply NEAPP-AM treatment to intraperitoneal micro dissemination which is difficult to control with conventional treatments.

## Conclusion

In conclusion our results suggest that NEAPP-AM can induce apoptosis in CCC while normal cells are less damaged. These findings indicate the possibility of being able to apply this technical modality intraperitoneally, and we are now trying to apply NEAPP-AM to I.P. therapy on a murine peritoneal metastasis model with EOCs including chemo-resistant cells. NEAPP-AM may be a promising new alternative for chemoresistant EOC treatment.

## Methods

### Cell culture

We used four ovarian cancer cell lines involving omentum-derived human fibroblastic cells (OHFC) and human peritoneal mesothelial cells (HPMC). TOV21G, ES-2, and SKOV3 were purchased from the American Tissue Culture Collection. The NOS2 cells, derived from serous EOC, were established in our institute. We described the details in our previous reports (Kajiyama et al. 2007a). OHFC and HPMC were isolated from pieces of human omentum. The tissues were obtained from consenting patients undergoing abdominal surgery at our institute (Kajiyama et al. 2007b). The study was approved by the institutional ethics committee (Approval number: 1234, Ethics Committee of Nagoya University). All cell lines were maintained in RPMI-1640 (Sigma, St. Louis, MO, USA) supplemented with 10% fetal bovine serum (FBS) and penicillin-streptomycin at 37°C in a humidified atmosphere of 5% CO_2_.

### Experimental system specification and production of nonequilibrium atmospheric pressure plasma-activated medium (NEAPP-AM)

**Figure 7:**
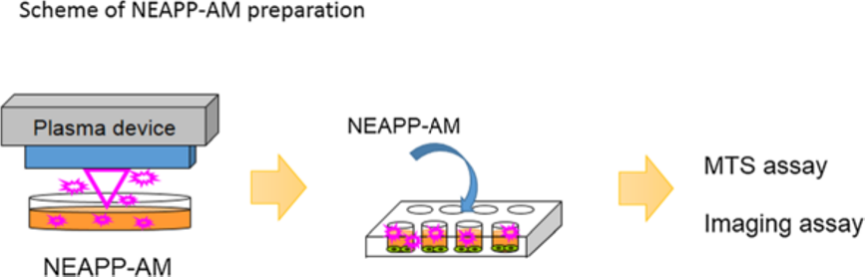
**Scheme of procedure to produce nonequilibrium atmospheric pressure plasma-activated medium.**

The details of this experimental NEAPP system were previously described (Iseki et al. 2012). Discharge conditions were in argon gas (2 standard liters/ min; slm) excited by applying 10 kV of a 60-Hz commercial power supply to two electrodes with a distance of 8 mm. In brief, NEAPP with an ultra-high electron density (approximately 2 × 10^16^ cm^-3^) was provided with an ultra-high O density estimated at around 4 × 10^15^ cm^-3^ (Iseki et al. 2012). Furthermore, the generation of ROS, such as hydroxyl radicals, singlet oxygen radicals, nitrogen oxide, and nitrogen, was confirmed by optical emission spectroscopy. We exposed the above NEAPP to RPMI-1640 without FBS separately from the cells, which is designated as ‘nonequilibrium atmospheric pressure plasma-activated medium (NEAPP-AM)’. A schema of this procedure is shown in Figure 7. The separated distance between the plasma source and medium (L) is critical to reproduce consistent data, so all experiments were performed under two set conditions: L = 15 and 25 mm, indicated as NEAPP-AM (15) and NEAPP-AM (25), respectively. The cytotoxic effect of NEAPP-AM (15) was suggested to be stronger than NEAPP-AM (25). NEAPP-AM (25) was used for cell viability assays, while NEAPP-AM (15) was used for cell imaging assays. The duration of plasma treatment ranged from 0 to 8 minutes. Six milliliters of RPMI-1640 medium was placed in a 60-mm dish. The center of each 60-mm dish was treated several times (1, 3, 5, and 8 min) with NEAPP fixed above the dish at a single point, referred to as NEAPP-AM-1, -3, -5, and -8, respectively. NEAPP exposure can cause changes to media in pH or temperature. We have referred to them in our previous manuscript and they were almost negligible (Iseki et al. 2012). ROS in medium were generated homogeneously due to the convective flow by gas or sufficient immixture before the addition to cells.

### Cell viability assay

The effect of NEAPP-AM on the viability of cells was measured with the Aqueous One Solution Cell Proliferation Assay kit (Promega, Madison, WI, USA), according to the manufacturer’s instructions. Absorbance was then measured at 490 nm with a microplate absorbance reader (ELx808; Bio Tek, U.S.A.). The cells were plated in 96-well plates at a density of 1 × 10^4^ cells per well in 100 μL of complete culture medium. When the assay included OHFC or HPMC, collagen-coated plates were used for all cell lines. The next day, cells were treated with NEAPP-AM (25) (1-8 min/6 mL) for 24 hrs. Experiments were performed in triplicate.

### The influence of modulation of reactive oxidative species (ROS) by N-acetyl cysteine and L-buthionine-[S, R]-sulfoximine

N-acetyl cysteine (NAC, Sigma-Ardrich, St. Louis, MO, USA), known as an intracellular ROS scavenger, was used to attenuate the effect of ROS. Alternatively, L-buthionine-[S, R]-sulfoximine (BSO, Sigma-Aldrich, St. Louis, MO, USA) , an inhibitor of GSH synthesis, was used to enhance the effect of ROS. It is known that GSH is one of the most abundant and effective components of the defense system against free radicals, including ROS (Jefferies et al. 2003). The compounds NAC and BSO were added to cells at a final concentration of 4 and 2 mM, respectively. The required volume of each drug was added directly to complete the cell culture medium 1 hrs before NEAPP-AM treatment and NEAPP-AM to achieve the desired final concentrations, respectively. Cell viability was examined with the “cell viability assay”.

### TUNEL assay

Apoptotic cells were identified using the *In Situ* Cell Death Detection Kit, Fluorescein (Roche Applied Science, Mannheim, Germany), according to the manufacturer’s instructions. TOV21G and SKOV3 cells (2 × 10^4^/well) were seeded in a collagen-coated 8-well cover glass, incubated for 24 hrs, and then treated with NEAPP-AM (15)-5 or serum-free medium as a control. After 4 hrs of incubation, cells were fixed with 4% paraformaldehyde and the TUNEL reaction mixture was added. After being incubated in a chamber for 60 min at 37°C, cells were observed with a fluorescence microscope. This experiment was repeated at least three times.

### Statistical analysis

Data are presented as means ± SD from at least three independent experiments. Statistical analysis of the data between two gropes was performed using Student’s *t-*test and statistical analysis between more than two gropes was performed by Dunnett’s test or Tukey-Kramer test Differences between groups were considered significant at *P* < 0.05.

## Abbreviations

EOC: Epithelial ovarian cancer
CCC: Ovarian clear cell carcinoma
ROS: Reactive oxygen species
NEAPP: Nonequilibrium atmospheric pressure plasma
OHFC: Omentum-derived human fibroblastic cells
HMPC: Human peritoneal mesothelial cells
FBS: Fetal bovine serum
NEAPP-AM: Nonequilibrium atmospheric pressure plasma-activated medium
GPx3: Glutathione peroxidase
*GLRX*: Glutaredoxin
SOD2: Superoxide dismutase.
